# Evaluation and modulation of DNA lesion bypass in an SV40 large T antigen‐based *in vitro* replication system

**DOI:** 10.1002/2211-5463.13099

**Published:** 2021-02-25

**Authors:** Zoltán Szeltner, Ádám Póti, Gábor M. Harami, Mihály Kovács, Dávid Szüts

**Affiliations:** ^1^ Institute of Enzymology Research Centre for Natural Sciences Budapest Hungary; ^2^ ELTE‐MTA “Momentum” Motor Enzymology Research Group Department of Biochemistry Eötvös Loránd University Budapest Hungary; ^3^ MTA‐ELTE Motor Pharmacology Research Group Department of Biochemistry Eötvös Loránd University Budapest Hungary

**Keywords:** *in vitro* replication, lesion bypass, next‐generation sequencing, nucleotide excision repair, T antigen, translesion synthesis

## Abstract

DNA damage removal by nucleotide excision repair (NER) and replicative bypass via translesion synthesis (TLS) and template switch (TSw) are important in ensuring genome stability. In this study, we tested the applicability of an SV40 large T antigen‐based replication system for the simultaneous examination of these damage tolerance processes. Using both Sanger and next‐generation sequencing combined with lesion‐specific qPCR and replication efficiency studies, we demonstrate that this system works well for studying NER and TLS, especially its one‐polymerase branch, while it is less suited to investigations of homology‐related repair processes, such as TSw. Cis‐syn cyclobutane pyrimidine dimer photoproducts were replicated with equal efficiency to lesion‐free plasmids *in vitro*, and the majority of TLS on this lesion could be inhibited by a peptide (PIR) specific for the polη‐PCNA interaction interface. TLS on 6–4 pyrimidine–pyrimidone photoproduct proved to be inefficient and was slightly facilitated by PIR as well as by a recombinant ubiquitin‐binding zinc finger domain of polη in HeLa extract, possibly by promoting polymerase exchange. Supplementation of the extract with recombinant PCNA variants indicated the dependence of TLS on PCNA ubiquitylation. In contrast to active TLS and NER, we found no evidence of successful TSw in cellular extracts. The established methods can promote *in vitro* investigations of replicative DNA damage bypass.

## Graphical abstract

The replication of photolesion‐containing plasmids was tested in an *in vitro* replication system using multiple detection methods. Polη‐driven translesion synthesis, which we successfully modulated by the addition of specific peptides and mutated recombinant PCNA, was mainly responsible for cyclobutyl pyrimidine dimer bypass, whereas TT(6‐4) lesions could only be processed by nucleotide excision repair. Template switching bypass was inefficient in the extracts.

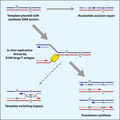

**Abbreviations**
AEBSF: (4‐(2‐Aminoethyl) benzene sulfonyl fluoride hydrochloride
CPD: cyclobutyl pyrimidine dimer
CTD: C‐terminal domain
HMW: high molecular weight
HPLC: high‐performance liquid chromatography
MCS: multiple cloning site
NER: nucleotide excision repair
NGS: next‐generation sequencing
NLS: nuclear localisation signal
NTP: ribonucleoside triphosphate
dNTP: deoxyribonucleoside triphosphate
PIP: PCNA‐interacting peptide
PIR: PCNA‐interacting region
qPCR: quantitative PCR
RF (I, II etc.): replicative form
RIR: REV1‐interacting region
SV40: simian virus 40
TLS: translesion synthesis
TSw: template switching
UBZ: ubiquitin‐binding zinc finger
UV: ultraviolet

Thousands of DNA lesions are generated by endogenous and exogenous agents in a day in mammalian cells [1]. Exposure to the ultraviolet component of sunlight can induce dimer formation of adjacent pyrimidine bases in DNA resulting in a number of forms of ultraviolet (UV) lesions, of which cis‐syn cyclobutane pyrimidine dimers (CPD) are the most frequent [2, 3]. CPD lesions present a significant contribution to UV‐induced mutagenesis, presumably because they are removed by nucleotide excision repair (NER) inefficiently [4]. If not eliminated, the lesions may stall replication forks. Stalled replication can be rescued by DNA damage tolerance mechanisms: translesion synthesis (TLS), or homology‐dependent template switching (TSw) in order to avoid replication fork collapse that leads to double‐strand break formation [5]. In TLS, specialised DNA polymerases replace the replicative DNA polymerase (polδ or polε) and synthesise the new DNA strand along the damaged template, while in TSw the template sequence information at the lesion site is obtained from the nascent strand of the intact sister chromatid [6]. Several TLS polymerases may bypass a particular type of lesion [7]; for example, polymerase η was shown to be able to bypass TT(CPD) lesions on its own *in vitro* [8] and probably in human cells as well [9]. In contrast, experiments in knockout cell lines demonstrated that other lesions such as the T‐T 6‐4 pyrimidine–pyrimidone (TT(6‐4)) photoproduct or BP‐G (trans‐BPDE‐N2‐dG) are bypassed by two‐step TLS requiring the contribution of more than one polymerase, usually a Y family polymerase with an inserter function (polη, polκ or polι) and the B family polζ as a general extender [9, 10]. The polζ4 complex (REV3‐REV7‐POLD2‐POLD3) was found to be the active general extender, sharing its POLD2 and POLD3 subunits with polδ [11, 12, 13].

TLS is regulated at the level of polymerase recruitment. Y family TLS polymerases can be recruited to the site of DNA lesions by binding to monoubiquitylated PCNA (proliferating cell nuclear antigen) through their evolutionarily conserved ubiquitin‐binding domains [14, 15]. However, in chicken and mouse cells, TLS has also been observed in the absence of PCNA ubiquitylation [14, 15, 16]. REV1, the fourth Y family TLS polymerase, was shown to bind directly to PCNA independent of its ubiquitylation status, but PCNA monoubiquitylation slightly increased its binding in a study [17]. REV1 was shown to be able to bind all of polymerases η, ι, κ and ζ at its C‐terminal CTD domain, coordinating two‐step TLS involving an insertion step by polη/ι/κ and an extension step by polζ [18, 19, 20].

*In vitro* replication experiments using extracts of simian virus 40 (SV40)‐infected cells [21], or using discrete components as in SV40 DNA or plasmids containing the SV40 origin of replication as templates, SV40 large T antigen as replication initiator protein and cytosolic mammalian cellular extracts as the source for the main chain‐elongating factors, have been used since the mid‐1980s and proved to be useful in elucidating the mechanism of DNA replication [22, 23, 24, 25, 26, 27]. The *in vitro* replication assays generally contained buffered cytosolic extracts of mammalian cells, the four ribonucleoside triphosphates and deoxyribonucleoside triphosphates, and ATP‐regenerating components. Plasmid DNA undergoes multiple rounds of DNA replication upon the addition of purified viral or recombinantly expressed T antigen and forms monomeric rings (supercoiled replication form (RF) I and relaxed/nicked circular RF IV/RF II replication products) as well as complex high molecular weight (HMW) structures [24].

Various plasmid‐based assays have been designed for studying replication. The chemical mutagen N‐2‐acetylaminofluorene [28], as well as UV photolesions, was examined using *in vitro* assays [29, 30, 31]. A particularly powerful method was devised by Lawrence and colleagues for studying DNA damage tolerance involving plasmids with photoproduct lesions incorporated into each strand in a staggered arrangement [32]. The methodology was later used in yeast studies [33] and adapted to DT40 chicken cell lines using a plasmid that is capable of episomal replication. The photoproducts were placed opposite mismatched bases, which allowed the detection, in recovered replicated copies, of TLS or TSw bypass in our laboratory [16, 34]. Although *in vivo* studies where synthetic lesions are introduced into the chromosome are now gaining ground [6], several advantages of *in vitro* assays remain. They permit straightforward interference studies for studying protein–protein interactions in DNA damage tolerance and repair, with exogenous materials (peptides, small molecular weight inhibitors, recombinantly expressed protein domains, etc.) simply added to the extracts. In addition, this approach allows the quantitative addition of external agents in titration studies and the investigation of agents that are toxic to cells or cause stress responses upon transfection.

In the present study, we combined the plasmid‐based in‐cell lesion‐bypass methodology [16, 33, 34] and the T antigen‐based *in vitro* replication system and checked the applicability of this system for producing truly replicated material and lesion bypass. We also sought to investigate whether lesion‐bypass routes (TLS and TSw) can be modulated using external agents and by interference with PCNA ubiquitylation. Although detection of true TSw events was hindered by a PCR artefact, we found that TLS (especially on TT(CPD) lesion) can be efficiently studied and modulated with specific peptides and various forms of PCNA.

## Materials and methods

### Plasmids with TT(CPD) and TT(6‐4) UV photoproducts for *in vitro* replication

**Fig. 1:**
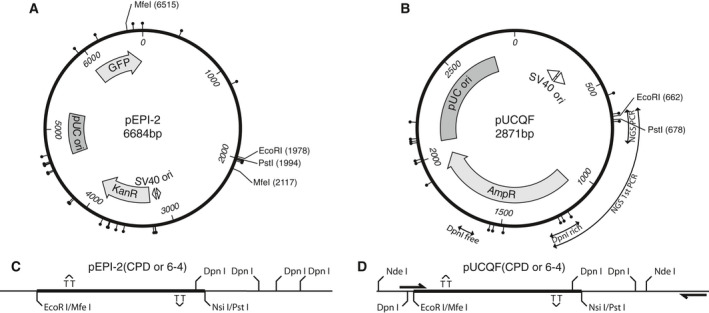
Plasmids used for the *in vitro* replication system. (A) pEPI‐2 (6684 bp) and (B) pUCQF (2871 bp) with SV40 replication origin (ori) between bp 3055–3190 and 253–388, respectively. PCR amplicons used for replication efficiency measurements (DpnI rich, DpnI free) as well as the first long amplicon and the final short amplicon used for NGS are shown on (B). (C, D) Oligonucleotides bearing T‐T photoproduct lesions on each strand in a staggered orientation were cloned between EcoRI and PstI sites on pEPI‐2 (C) or pUCQF (D). Primers for the final NGS amplicon are indicated (D, not to scale).

The episomally replicating pEPI‐2 plasmid (6684 bp, Fig. 1A) was created by a minimal modification of pEPI‐1 [35] at its multiple cloning site (MCS) to allow for the insertion of lesion‐containing oligonucleotides: the pEPI‐1 MCS was cut with EcoRI and BamHI, and KpnI, PstI and SpeI restriction sites were inserted using the partially complementary oligonucleotides AATTCGGTACCCTGCAGACTAGTG and GATCCACTAGTCTGCAGGGTACCG. pUCHSO (2880 bp) bearing an SV40 replication origin was obtained from EURx (Gdansk, Poland, Cat No: E5800‐02) and modified by eliminating NsiI (436), PstI (444) and EcoRI (479) sites from its MCS and inserting a cloning cassette (DpnI‐EcoRI‐XbaI‐PstI‐DpnI) into the unique NdeI site (692), producing pUCQF (2871 bp) (Fig. 1B). Two partially complementary oligonucleotide pairs containing either TT(CPD) or TT(6‐4) photoproducts were synthesised as before [16, 34] and ligated into the EcoRI and PstI sites of pEPI‐2 and pUCQF plasmid with a two‐step procedure as described [33] in order to place the lesions into both strands of the plasmid in staggered arrangements 28 bp from each other opposite CG mismatches (Fig. 1C,D). Lesion‐free control oligonucleotides with the same sequence were also ligated. The pUC8‐4 (Ori–) control plasmid without a functional SV40 origin was also purchased from EURx.

### Preparation of cytosolic extracts

Cytosolic extract was prepared from proliferating human HeLa, TK6 wild‐type and TK6 *XPA^−/−^* [36] cells, cultivated as monolayers (HeLa) or as suspension cultures (TK6). HeLa cells were obtained from Sigma‐Aldrich, St. Louis, MO, USA; the TK6 lines were a gift from Shunichi Takeda at the TK6 mutant consortium (http://www.nihs.go.jp/dgm/tk6.html). After reaching confluence on twelve 140‐mm plates (Nunc, cat No‐168381), HeLa cells were washed twice with PBS and then washed twice with hypotonic buffer containing 20 mm HEPES‐KOH, pH 7.6, 5 mm potassium acetate, 1.5 mm MgCl_2_, 0.5 mm DTT, 1 mm AEBSF (4‐(2‐aminoethyl) benzenesulfonyl fluoride hydrochloride) at 4 °C. Swollen cells were harvested by a scraping into two 1‐mL Dounce homogenisers and disrupted by 20 strokes with a tight‐fitting pestle. NaCl (5 m) was added to the material adjusting it to 200 mm, and after incubation on ice for 10 min, the nuclei were separated by centrifugation (2 min at 1000 ***g***). The supernatant containing the cytosolic fraction was then ultracentrifuged (50 000 ***g*** for 30 min). The supernatant was dialysed against storage buffer (10 mm HEPES‐KOH, pH 7.6, 1 mm DTT, 0.1 mm EDTA, 10 mm KCl, 10% glycerol (v/v) for 2 h on ice using a Slide‐A‐Lyser Dialysis Cassette (Thermo Fisher Scientific, Waltham, MA, USA, Cat. No 66203), followed by centrifugation (50 000 ***g*** for 60 min). The protein concentration of the clear supernatant was determined with Bradford assay. The material was frozen in small drops in liquid nitrogen and stored at −80 °C until used. When preparing extracts from TK6 cells grown in suspension culture, cells (2.5 × 10^8^) were centrifuged at 400 ***g*** for 10 min, washed twice with PBS, cooled down on ice (2 min) and were suspended in 0.8 mL hypotonic buffer followed by cell disruption and the same further steps as with HeLa cells. HeLa cellular extract was also obtained from a commercial source (EURx, Cat No: E8050). Nuclear extracts were prepared by adding NaCl solution to the separated nuclei up to 0.4 m followed by incubation at 4 °C in a rotary shaker for 1 h then centrifugation at 50 000 ***g***. The supernatant was also dialysed against the storage buffer and frozen in small aliquots.

### *In vitro* replication assays

T antigen was obtained from EURx (Cat No: E5800‐02). Plasmids with or without the cloned UV photolesions were replicated for 3 h at 37 °C in 10 µL reaction mixtures containing (final concentrations) 30 mm HEPES (pH 7.6), 7 mm MgCl_2_, 0.5 mm DTT, 4 mm ATP, 100 μm each of dATP, dGTP, dTTP, dCTP, 80 μm each of CTP, GTP, UTP, 40 mm phosphocreatine, 0.4 unit creatine phosphokinase, 30 ng plasmid DNA, 0.4 μg T antigen, 30 μg cytosolic extract. Reactions were terminated by the addition of SDS/EDTA to 0.75% (w/v)/22.5 mm final concentrations. DNA was deproteinised by incubation with proteinase K (1 µL of 10mg·mL^−1^) at 55 °C for an hour. Digested proteins were precipitated with ammonium acetate (1.25 m) at 4 °C and centrifuged (20 000 ***g*** for 10 min). In order to precipitate DNA, glycogen (1 µL of 10 mg·mL^−1^) and 2‐propanol (0.8 volume) were added to the supernatant, mixed and then centrifuged (20 000 ***g*** for 30 min). The pellet was washed with 70% EtOH, dried and routinely dissolved in the reaction mixture prepared for DpnI digestion and digested at 37 °C for 60 min with 10 U DpnI in NEB4 buffer (New England Biolabs, Ipswitch, MA, USA) supplemented with NaCl to 200 mm final concentration. The DNA was precipitated with 2‐isopropanol and washed with 70% EtOH as above and dissolved in 20 µL water.

When replication reactions were analysed with gel electrophoresis followed by autoradiography, dCTP was used at 50 µm concentration and α32P‐dCTP (Hartmann Analytic, Braunschweig, Germany) was also included in the reaction at about 100 kBq activity. Agarose gel electrophoresis was done in TBE (Tris/borate/EDTA) buffer. Dried gels were visualised using a phosphor screen and a Molecular Imager FX instrument, and the signal was quantified using the image lab software (Bio‐Rad Laboratories, Hercules, CA, USA).

### Testing plasmid replication efficiency

Samples from DpnI‐treated T antigen‐driven plasmid amplification reactions were subjected to quantitative PCR (qPCR) with a CFX96 instrument (Bio‐Rad), using a primer pair named pUCQF0 (CTTCCTGTTTTTGCTCACCC and GTTCTTCGGGGCGAAAACT) amplifying a DpnI free region (1609–1680) of pUCQF or pUC8‐4, as well as primer pair pUCQF3 (CTGAATGAAGCCATACCAAACG and TTTGCGCAACGTTGTTGCCATT) amplifying a DpnI site‐rich region of the beta lactamase gene (1165–1297, three DpnI sites) on the plasmids. 10 µL qPCR assays were run using PerfeCTa SYBR Green FastMix (Quanta) master mix. The amount of template was quantified using standard curves obtained with pure pUCQF plasmid. The pUCQF3/pUCQF0 product ratio was used to calculate the percentage of DpnI‐resistant, replicated plasmids.

### Determining bypass outcomes

In direct transformation experiments, DpnI‐digested replication mixtures from TT(CPD) and TT(6‐4) bypass studies were transformed to TOP10 competent *E. coli* cells followed by plasmid isolation from individual colonies and Sanger sequencing.

In order to determine bypass outcomes by next‐generation sequencing (NGS)‐based amplicon sequencing, first a longer 652‐bp region of the pUCQF replication product (covering the sites of the lesions and five interspersed DpnI sites) was amplified by PCR using Pfu Turbo (Agilent Technologies, Santa Clara, CA, USA) and primers 5′‐AGAACTCATATGGATCGAATTGTC‐3′ and 5′‐GTTCTTCGGGGCGAAAACT‐3′, then a 239‐bp subregion covering the lesions and the flanking DpnI sites was amplified with primers that contained unique barcodes in each primer for each experiment. The resulting amplicons were mixed and subjected to library preparation and sequencing using the Illumina HiSeq platform with 2 × 150 bps paired‐end reads at Novogene, China. The forward and reverse reads were trimmed using Trimmomatic, paired using FLASH [37] and aligned against the reference sequence using Bowtie2 [38]. Frequencies of sequences referring to possible damage bypass events were extracted using standard Unix command‐line tools and visualised in R.

For assaying lesion bypass using sequence‐specific qPCR, control plasmids for allele‐specific qPCR representing the three typical replicative bypass sequence products (GC/GC representing homology‐dependent repair, TT/GC representing TLS at the top lesion and GC/TT representing TLS at the bottom lesion) and two additional control plasmids containing a gap (two bp deletion) at the top lesion site (GAP/GC) or at the bottom site (GC/GAP) were generated by cloning the corresponding duplex oligonucleotides to the EcoRI‐PstI sites of pUCQF. Different PCR primers ending at the lesion sites with specificity to the above templates were also synthesised (Fig. 4G), and standard curves for each unique template and its cognate sequence‐specific primers were obtained using 10 µL reactions containing 0.4 µm of the primers, 2 µL template of various dilutions (from ~ 20 ng·µL^−1^ to 2 × 10^−8^ ng·µL^−1^) and 5 µL 2X PerfeCTa SYBR Green FastMix mastermix (Quantabio, Beverly, MA, USA). The specificities of each of the five lesion‐specific primer pairs on all templates were also tested (Table S1). Only GC/GC‐ and TT/GC‐specific primer pairs showed significant cross‐specificity to the PCR‐generated GAP/GC, while primer pairs detecting true lesion‐bypass outcomes (GC/GC, GC/TT, TT/GC) showed negligible cross‐specificities. All observed promiscuities were considered in the calculations.

When determining replication outcomes, we used the same 652 bp PCR product template that was used for generating materials for NGS, diluted to keep Cq values between 10 and 20. Cq values obtained from the reactions with each of the individual lesion‐specific primer pairs were fitted to the standard curves and the amounts of individual bypass products were determined and plotted as percentages.

### Assaying NER in cellular extracts

Replication reactions using lesion‐free, TT(CPD) and TT(6‐4) containing plasmids were executed as in above with or without T antigen and without DpnI digestion. A 652‐bp PCR product was obtained as above and then purified by agarose gel electrophoresis. The purified material was used for Sanger sequencing and for qPCR with primers (5′‐TTAGGCCATATGGATCGAATTGT‐3′ and 5′‐TAGCTTGGGGCTGGCTTAACTATG‐3′) to the 5′ and 3′ ends of the lesion site using CFX96 instrument (Bio‐Rad). Melting curves were recorded by the instrument at the end of run. Extracts from TK6 *XPA* *^−/−^* cells (30 ng per reaction) were supplemented with 1 ng pure XPA protein where indicated.

### Generation of PCNA variants for interference studies

Wild‐type human PCNA was produced by amplifying the coding region of the gene using primers 5′‐TAGGTGATCATATGTTCGAGGCGCGCCTGGT‐3′ and 5′‐CAGGATCCCTAAGATCCTTCTTCATCCTCGATCTTG‐3′ with NdeI and BamHI sites (underlined) and cloning the PCR product in frame with an N‐terminal 6× His‐tag into pET15b. The protein was produced in *E. coli* BL21 STAR (DE3) and purified by metal chelate affinity chromatography on a Ni‐NTA superflow column (Qiagen, Hilden, Germany) according to the manufacturer’s instructions to about 90% purity, followed by size‐exclusion chromatography on a Superose 12 column (GE Healthcare, Chicago, IL, USA). The purified protein was dialysed against 20 mm K‐HEPES pH 7.6, 10 mm KCl, 0.5 mm DTT, 20% glycerol, frozen in liquid nitrogen and stored at −80 °C in small aliquots. The K164R mutation was inserted using the QuikChange Lightning Site‐Directed Mutagenesis Kit (Agilent #210518) with primers 5′‐GTAATTTCCTGTGCAAGAGACGGAGTGAAATTTTC‐3′ and 5′‐GAAAATTTCACTCCGTCTCTTGCACAGGAAATTAC‐3′. The protein was expressed and purified as wild‐type PCNA.

The PCNA‐Ub fusion sequence was amplified using primers 5′‐TAGGTGATCATATGTTCGAGGCGCGCCTGGT‐3′ and CAGGATCCCTACCTCAGGCGCAGCACCAGATG from our published eukaryotic vector [39] that contained the PCNA gene with a C‐terminally fused ubiquitin. After cloning the PCR product into the NdeI‐BamHI sites of pET15b expression vector, the recombinant protein was expressed in *E. coli* BL21 STAR (DE3) with a N‐terminal His‐tag and purified as the other PCNA variants.

### Generation of recombinant XPA

Human XPA (transcript variant 1) was produced by amplifying the coding region of the gene using cDNA template from OriGene (Rockville, MD, USA; CAT#: SC319524) and primers GATTATATACATATGGCGGCGGCCGACG and ATTAGGATCCTCACATTTTTTCATAAGTCAGTTCATGGCCAC with NdeI and BamHI sites (underlined) and cloning the PCR product in frame with an N‐terminal 6 x His‐tag into pET15b. The protein was expressed in Rosetta (DE3)pLyS as described [40]. XPA was produced mostly in inclusion bodies. *E. coli* cells from 1 L culture were disrupted by sonication in buffer of 50 mm phosphate, 300 mm NaCl, 2 mm DTT, Protease Inhibitor Cocktail (Roche, Basel, Switzerland), 5% glycerol, pH 7.4. The sonicated material was centrifuged (22 000 ***g*** for 10 min), and the pellet was washed three times. The washed insoluble material was successfully renaturated by solubilising inclusion bodies in 6 m guanidine/HCl, 20 mm Tris/HCl, 200 mm NaCl, 0.5 mm DTT, 1 mm 4‐(2‐aminoethyl) benzenesulfonyl fluoride hydrochloride, 10% glycerol, pH 7.6. After 2‐h incubation at room temperature with gentle agitation in a flask, the 60 mL material was abruptly diluted with 800 mL renaturation buffer (20 mm Tris, 200 mm NaCl, 0.5 mm DTT, 10 µm ZnCl2, 10% glycerol, pH 7.6) and incubated overnight at 4 °C. After centrifugation, the clarified supernatant was supplemented with 10 mm imidazole, NaCl was adjusted to 400 mm and the pH to 8.0, and the material was applied to a 3 mL Ni‐NTA superflow column (Qiagen) and then washed with 15, 30 and 50 mm imidazole in the same buffer. More than 95% pure protein could be eluted with buffer containing 200 mm imidazole. The material from Ni‐NTA was concentrated to 2 mg·mL^−1^ and further purified/tested by size‐exclusion chromatography on a Superose 6 column (GE Healthcare) in a buffer of 10 mm K‐HEPES pH 7.6, 150 mm NaCl, 0.5 mm DTT, 0.1 mm EDTA, 10% glycerol. No aggregated material was detected and the material eluted at a volume corresponding to monomeric XPA. The pure material was buffer exchanged with the renaturation buffer with only 20 mm salt (KCl), concentrated to 0.3 mg·mL^−1^, frozen in liquid nitrogen and stored at −80 °C in small aliquots.

### Other materials used for interference studies

The DNA sequence corresponding to the ubiquitin‐binding zinc finger (UBZ) peptide (domain) of polη was synthesised using three primers of 5′‐GGGAGCCCATATGGCTGCTGAAGACCAGGTTCCGTGCGAAAAATGCGGTTCTCTGGTT‐3′, 5′‐GAAAAATGCGGTTCTCTGGTTCCGGTTTGGGACATGCCGGAACACATGGACTACCACTTC‐3′ and 5′‐GGAGCCGGATCCTTAAGATTTCTGCAGTTCCAGAGCGAAGTGGTAGTCCATGTGTTC‐3′ with a two‐step PCR filling/amplification reaction. The PCR amplified gene was cloned into pET15b expression vector, and the protein domain was expressed in *E. coli* BL21 STAR (DE3) with a N‐terminal polyhistidine tag, then purified by Ni‐NTA chromatography. The polyhistidine tag was removed by on‐column digestion with thrombin. The mature protein with an extra GSHM peptide on the N terminus was further purified by size‐exclusion chromatography on a Superose 12 column (GE Healthcare). The eluted protein was dialysed against the storage buffer (10 mm HEPES‐KOH, pH 7.6, 1 mm DTT, 0.1 mm EDTA, 10 mm KCl, 20% glycerol) and stored at −80 °C in small aliquots.

The clone coding for a truncated polζ2 complex pET‐Duet‐1‐(His‐Rev7(R124A)Rev3(1847‐1898)) was a generous gift from Dr. Kodai Hara (SPS, University of Shizuoka, Japan). This clone made possible the co‐expression and copurification of the polyhistidine‐tagged REV7 protein in complex with the truncated REV3 protein as found in [41]. The complex was expressed in *E. coli* BL21 STAR (DE3) and purified by metal affinity chromatography.

The RIR peptide (REV1 interacting region, QSTGTEPFFKQKSLLL) corresponding to the REV1 binding domain of polη and the PIR peptide (PCNA‐interacting region), KRPRPEGMQTLESFFKPLTH) that contains the PIP motif (PCNA‐interacting peptide, underlined) and a part of the nuclear localisation signal (NLS) sequences plus the last four C‐terminal amino acids of polη were chemically synthesised (by Luiz Juliano at UFSB, São Paulo, Brazil) and high‐performance liquid chromatography‐purified. As controls, ‘inert peptide 1’ (bradykinin, RPPGFSPFR) and ‘inert peptide 2’ (salmon calcitonin, CSNLSTCVLGKLSQELHKLQTYPRTNTGSGTP) were used.

### Interference assays

When testing the effect of different recombinant PCNA variants (in HeLa and TK6 cell extracts), extracts (30 µg) were preincubated with fixed concentrations (50 µm) of the recombinant PCNA proteins for 30 min at room temperature in order to allow time for equilibration with endogenous PCNA. In separate tubes, T antigen was also preincubated with the lesion‐containing DNA and with the other components of the replication reaction to aid the assembly of the hexameric form of T antigen on the SV40 origin of the vector. UBZ peptide (220 µm) and the truncated polζ2 protein (100 µm) were also preincubated with the extract. Uniting the contents of the two tubes initialised the replication/repair reaction. PIR was tested at a fixed 100 µm concentration in HeLa extract and at a range of concentrations in TK6 extracts. RIR peptide was tested at 100 µm concentration in all extracts.

RecQ family helicase interference was studied using the pUCQF (CPD) construct. Full‐length BLM helicase (comprising 1417 amino acids) and a truncated BLM variant (residues 642‐1191) were recombinantly expressed in yeast and purified to homogeneity as described earlier [42, 43, 44] RecQ was expressed in *E. coli* and purified as described [45, 46]. The helicases were added to the replication reaction at two concentrations (40 and 400 nm). Lesion bypass was analysed by the qPCR method and by Sanger sequencing.

## Results and Discussion

### SV40 origin‐bearing plasmids with or without UV lesions can be efficiently replicated using recombinant T antigen in HeLa cellular extracts

Based on the classic experiments of Wobbe *et al*. [23, 24], we set up and tested an *in vitro* replication system in order to study lesion bypass in cellular extracts. Two plasmids of different size were created. We initially explored the use of a derivative of the large pEPI‐1 [35] plasmid possessing an SV40 origin, the 6684 bp pEPI‐2 (Fig. 1A). A shorter vector, pUCHSO (EURx), a derivative of pUC19 with an SV40 origin (253–388), was further modified in our laboratory to obtain pUCQF (Fig. 1B) to allow for the ligation of lesion‐containing oligonucleotides (see Materials and methods).

We ligated oligonucleotides containing UV photolesions TT(CPD) and TT(6‐4) into the two plasmids to introduce lesions to both strands in a staggered fashion (Fig. 1C,D) for the purpose of testing lesion bypass, as these lesions block T antigen‐driven replication [47]. In this arrangement, all successful replication products must result from the bypass of one lesion or the other. New DpnI sites were also introduced on each side of the oligo insertion sites to aid the elimination of nonreplicated DpnI‐sensitive material.

**Fig. 2:**
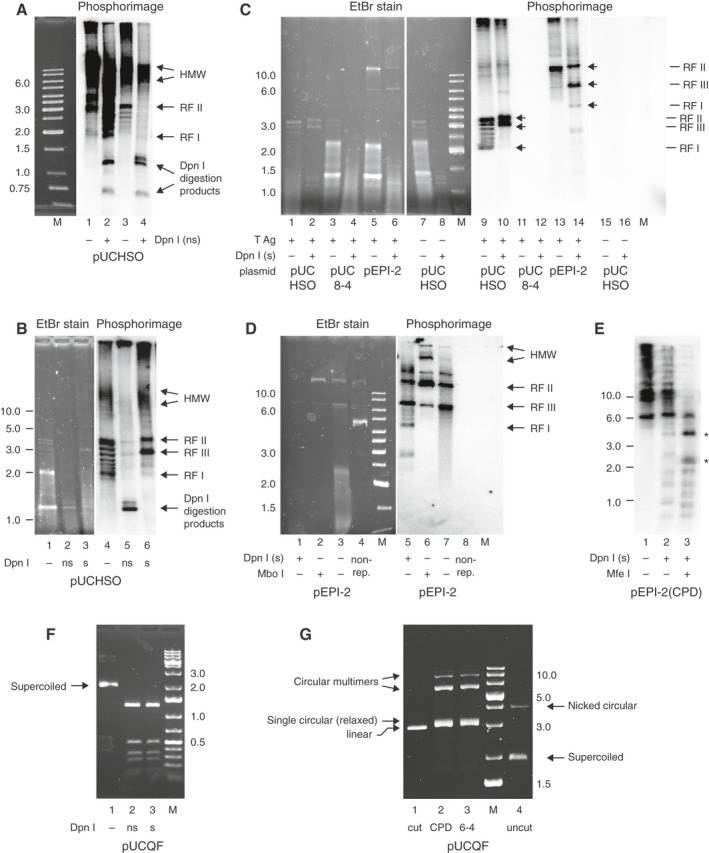
*In vitro* replication experiments. (A) Replication of pUCHSO plasmid DNA (2880 bp) using T antigen and HeLa extract from EURx (lanes 1–2) or EURx T antigen and our own HeLa extract (lanes 3–4). Different replicative forms (RF I: superhelical, RF II: nicked circular; fully relaxed circular RF IV replication products would migrate together with RF II) as well as HMW DNA are indicated. DpnI digests contained no extra salt (ns). (B) Supplementing DpnI digestion buffer with NaCl to 200 mm (s) inhibited the cleavage of hemimethylated replication products. (C) Comparing the replication of the smaller pUCHSO (with or without T antigen) and a larger pEPI‐2 plasmid with a control plasmid (pUC8‐4) without a functional SV40 origin. RF III: linear form of the plasmid. (D) DpnI and MboI digestions of DNA from pEPI‐2 replication. Lanes 4, 8 contain untreated pEPI‐2 template. (E) Replicated pEPI‐2(CPD) is resistant to DpnI digestion (lane 2) and can be cleaved into two fragments of 4398 and 2327 bp (indicated by asterisks) with MfeI. On (C‐E), DpnI digests were in the presence of 200 mm NaCl. (F) The pUCHSO variant pUCQF (lane 1, uncut bacterial prep) was digested with DpnI in the absence (ns) or presence (s) of 200 mm NaCl. (G) Gel images of linearised pUCQF plasmid (lane 1), ligated preparations containing the TT(CPD) and TT(6‐4) UV photoproducts as used for all subsequent experiments, and uncut bacterial plasmid preparation that was vortexed to show the position of nicked circular DNA (lane 4). The gel was run in the presence of EtBr to separate relaxed circular and nicked circular forms. Molecular size markers (M) are labelled; sizes are given in kb units. * are referred to as asterisks in the description of panel E.

We first tested the replication system using lesion‐free plasmids. DpnI only cuts dam‐methylated recognition sequences; therefore, the appearance of DpnI‐resistant DNA is indicative of replication *in vitro*. DpnI‐resistant materials of different size were obtained in both commercial and laboratory‐made HeLa extracts as shown on the phosphorimage representing DNA with incorporated 32P‐dCTP (Fig. 2A). The commercial extract and commercial T antigen combination (lanes 1 and 2) resulted in the highest level of replication, showing the highest amount of HMW, representing complex structures of plasmids that underwent multiple or partial rounds of DNA replication [24]. Monomeric rings also formed and were represented in a topoisomer ladder from relaxed RFs to the most supercoiled RF I, similar to the observations of Wobbe *et al*. [24]. Interestingly, DpnI cleaved a significant proportion of the radioactive material, especially the monomeric rings (Fig. 2A). These monomers may primarily represent plasmid molecules that underwent only one round of replication and thus remained hemimethylated, while the more strongly DpnI‐resistant HMW material is more likely to have replicated twice or more times and thus can be considered unmethylated. It has been shown that hemimethylated DNA can also be cleaved, albeit at a lower rate, by DpnI, and this activity is completely eliminated at higher ionic strength [24]. Therefore, we supplemented the enzyme buffer to 200 mm NaCl final concentration in our subsequent digestion studies (Fig. 2B). We compared the replication patterns of the products of the smaller pUCHSO and the larger pEPI‐2 to each other and to pUC8‐4 variant of pUCHSO that lacks a functional SV40 origin (Fig. 2C). The ethidium bromide‐stained gel showed slower migrating plasmid topoisomers following the addition of T antigen (lane 1 vs 7) and also showed that DpnI‐digested the original methylated template as well as the products of repair synthesis completely (e.g. nick translation products, if any) and left only the replicated material intact in the reactions that contained both an origin‐bearing template and large T antigen. All material was cleaved to small fragments in case of the pUC8‐4 reaction (lane 4), and when T antigen was omitted (lane 8), as expected in the lack of replication. Also, negligible amounts of radioactive products were synthesised in these reactions. Similar topoisomer ladders could be observed for both plasmids upon successful replication as in Fig. 2A, but the material was not cut by DpnI in the salt‐supplemented enzyme buffer (Fig. 2C lanes 9–10 and 13–14).

To further confirm plasmid replication, we digested the extracted replication mixtures with MboI, which cleaves double unmethylated (at least twice replicated) DNA, while it does not cleave the hemimethylated product or the starting template [48]. DpnI digestion eliminated the original methylated template, but the newly replicated (mostly hemimethylated) DNA remained intact and visible on both the ethidium bromide gel and the phosphorimage (Fig. 2D lanes 1 and 5). MboI as expected did not cleave the methylated template, and the newly synthetised material was added to it (Fig. 2D lane 2).

Interestingly, while intact pEPI‐2 DNA displayed a relaxed (RF IV/II) and a superhelical (RF I) form (Fig. 2D lane 4), the RF I form was missing from the replicated materials (lane 3), indicating that superhelicity was eliminated during incubation. Yet, replication also produced some radiolabelled RF I DNA in addition to the relaxed/nicked RF IV/II DNA (Fig. 2C,D). Most of the replicated material appears to be hemimethylated thus truly replicated DNA, where template DNA has undergone one round of replication and is cleavable with neither MboI nor DpnI.

Next, we tested the replication of pEPI‐2 plasmid that contained two TT(CPD) photoproducts cloned into each strand of the vector 28 bp from each other in staggered orientation. The reactions produced DpnI‐resistant material (Fig. 2E lanes 1 and 2), which could be cleaved into two fragments (marked by asterisks) with MfeI (Fig. 2E lane 3). The sizes of the fragments corresponded to the whole size of the plasmid and the relative intensity of the two bands was proportional to their length indicating that the whole length of the plasmid had been replicated, suggesting successful replicative lesion bypass.

### Efficient and error‐free *in vitro* bypass of CPD UV photolesions detected by next‐generation DNA sequencing

**Fig. 3:**
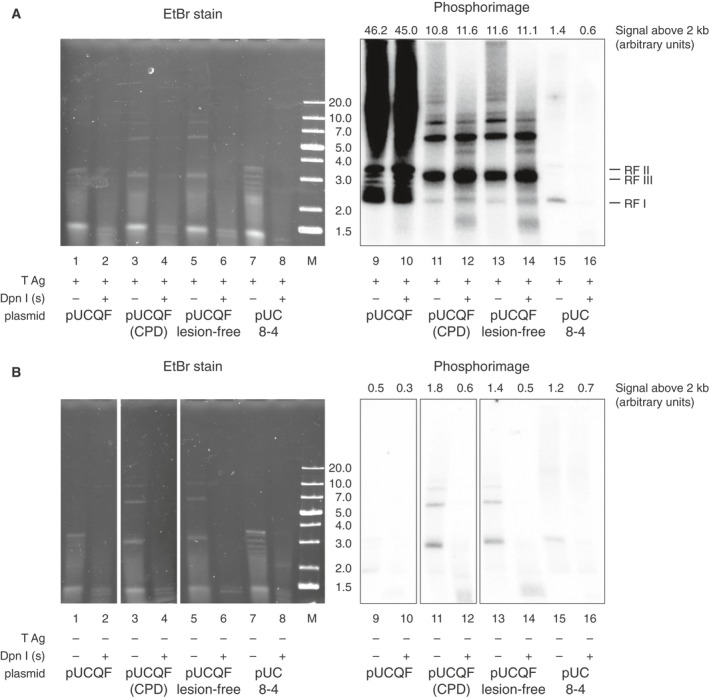
Replication of lesion‐containing templates. (A, B) The outcome of radioisotope‐labelled replication reactions containing the pUCQF control plasmid, ligated pUCQF preparations with a CPD lesion and a lesion‐free control, and the pUC8‐4 plasmid that lacks the SV40 replication origin. The reactions were performed in HeLa extract in the presence (A) or absence (B) of T antigen. DpnI digests were performed in the presence of 200 mm salt. The left and right panels are derived from the same gel after ethidium bromide staining and phosphor imaging of the dried gel, respectively. The subpanels in (B) were cut from one gel and rearranged for clarity. The phosphor images in (A) and (B) were exposed and scanned together. The quantification of the total signal above 2 kb (including the marked RF I form) is shown above the panel. M, molecular size marker labelled in kb units.

After successfully replicating the lesion‐free pUCHSO and pEPI‐2 plasmids and the CPD‐containing pEPI‐2 plasmid in HeLa extract, we investigated the replication of the pUCHSO variant pUCQF (Fig. 1B) that contained either TT(CPD) or TT(6‐4) lesions. All subsequent DpnI digests were performed in the presence of 200 mm NaCl, which did not inhibit the enzyme (Fig. 2F). The ligated lesion‐containing plasmid preparations did not contain any detectable amounts of linear or nicked circular DNA, though they did contain some circular multimers which unavoidably arise during the last, closing step of the sequential stoichiometric oligonucleotide ligation (Fig. 2G). These multimers are not expected to interfere with the PCR‐based methods for detecting sequence outcomes employed below. The ligated CPD‐containing and lesion‐free plasmids replicated with equal efficiency in the presence of T antigen, as judged by the incorporation of radiolabelled nucleotides into DpnI‐resistant products (Fig. 3A, lanes 12 and 14). We also observed some labelled replication products on the pUC8‐4 plasmid that lacks the SV40 origin (Fig. 3A) and on all tested plasmids in the absence of T antigen (Fig. 3B) but most of this signal was not DpnI‐resistant, suggesting partial replication.

**Fig. 4:**
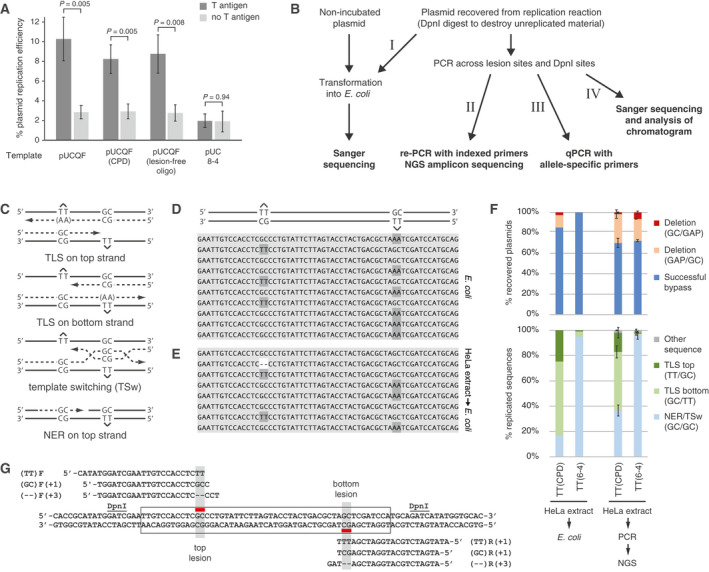
Replication of lesion‐containing plasmids. (A) Replication efficiency of the indicated control and lesion‐containing plasmids, measured by qPCR as the production of DpnI‐resistant material in the presence or absence of T antigen (TAg). The pUCQF8‐4 plasmid lacks the SV40 replication origin (Δori). The mean and SD of 3–4 measurements are shown; significant differences due to the presence of T antigen are indicated (*t*‐test). (B) Scheme for the evaluation methods (marked I–IV) for *in vitro* lesion bypass. (C) Schematic figure for monitoring lesion bypass. In case of error‐free TLS, AA is inserted against the lesions that are situated either in top strand (TT if sequenced from the left) or bottom strand (AA if sequenced from the left), while GC at both lesions represents the action of a process that copies information from the opposite strand as a possible result of TSw or NER. (D, E) Representative Sanger sequencing results from *E. coli* colonies following transformation with unreplicated pEPI‐2(CPD) (D) or with DpnI‐digested pEPI‐2(CPD) recovered from replication reactions (E). (F) Comparison of bypass assays on both CPD and 6‐4 photoproducts using *E. coli* transformation and Sanger sequencing (left two columns, using pEPI‐2) versus nested PCR and amplicon sequencing (NGS, right two columns, using pUCQF). Top panel, successful lesion bypass shown by the presence of nucleotides at the site of the lesion (see bottom panel), versus deletions at the lesion site, which signify gaps opposite the lesion in the replicated plasmid in the PCR approach (GAP). Bottom panel, sequence outcome categories in sequences showing successful bypass (TLS + TSw/NER). (G) Lesion‐specific primers for the qPCR‐based quantitation of major replication and lesion‐bypass products.

To establish a simpler method for obtaining information on the efficiency of the replication reactions, we carried out qPCR measurements on the DpnI‐digested replicated materials, comparing the product from a 133 bp amplicon with three DpnI sites to a control amplicon with no DpnI sites (see Materials and methods). The lesion‐free pUCQF as well as the plasmid construct with cloned lesion‐free oligos and the construct with cloned CPD‐containing oligos replicated with equal efficiency in HeLa extract that we prepared (8–10% of the template was replicated, Fig. 4A; the differences are not significant, *t*‐test). Interestingly, some replication was also evident in the absence of T antigen, at a similar level as was observed on the pUC8‐4 plasmid that lacks a functional SV40 origin (Fig. 4A). Some of this basal replicating capacity may be due to the presence of some replication initiation factors in the extracts [25]. However, as most of the labelled products that arose in the absence of T antigen were DpnI‐sensitive (Fig. 3B), it is also clear that the qPCR‐based assay gives a disproportionally high background signal.

The successful replication in HeLa extract suggested that lesions were bypassed by at least one of the possible lesion tolerance pathways. Fig. 4B shows a scheme that we followed in evaluation of lesion‐bypass deployments. The use of staggered lesions opposite mismatched bases makes it possible to discriminate between the mechanisms that use an alternative template (sister chromatid) versus TLS, where specialised polymerases directly copy the damaged strand. However, lesions can also be removed prior to replication by NER, which is also an error‐free process that uses the lesion‐free strand as template to replace the excised damaged section [49]. During correct TLS, AA is inserted opposite of a CPD lesion (TT is sequenced for the lesion on the top strand or AA on the bottom strand). Alternatively, the information of the nascent strand of the sister chromatid (TSw) or the opposite strand (NER) is used in the error‐free processes generating sequence outcomes with GC at the site of both lesions (Fig. 4C).

We established and tested a range of methods for measuring the outcome of lesion bypass. In our first approach (approach I on Fig. 4B), replication outcomes were determined by direct transformation of the DpnI‐resistant replication products into bacteria. As a control, we tested the transformation of a lesion‐containing plasmid, pEPI‐2(CPD), into *E. coli*. Many colonies formed with this undigested control material with TLS as the preferred (90%) bypass route (Fig. 4D, 10 colonies were sequenced) suggesting that *E. coli* has intrinsic pathways for bypassing CPD lesions. Indeed, DinB (DNA polymerase IV) was shown to be able to insert nucleotides opposite of a replication‐stalling N2‐dG lesion [50] and the CPD lesion was also bypassed in a relatively (93%) error‐free manner in an earlier study [51]. However, as expected, no colonies were formed when we transformed *E. coli* with pEPI‐2(CPD) previously digested with DpnI (in salt‐supplemented NEB4 buffer). Having established *E. coli*‐based controls, we next transformed *E. coli* with the DpnI‐digested reaction products of TT(CPD) and TT(6‐4) containing plasmids replicated in HeLa cell extract. Upon sequencing DNA from a total of 47 and 50 colonies, respectively, TLS was represented in 80% for CPD (Fig. 4E,F) and only in 6% (Fig. 4F) for TT(6‐4), and no mutagenic bypass events were detected (Table S2). TLS was more preferred on the bottom strand, which should be the leading strand if replication proceeded symmetrically from the origin located about 350 bp upstream (Fig. 1B). However, the strand bias may also be a result of difference in the sequence context around the lesion, shown to be important both the extent and the accuracy of TLS [52]. The transformation of bacteria with DpnI‐digested products of equivalent reactions in which T antigen was omitted produced no colonies, confirming that the assay detects true replication products. The results show that direct transformation of *E. coli* with the replication products is a viable, though inefficient method of assaying the outcome of lesion bypass *in vitro*. Moreover, this method may give misleading results if the *in vitro* replicated plasmid contains a postreplicative gap at the lesion, as in such cases *E. coli* would be able to fill in the gap using TLS on the DpnI‐resistant plasmid.

We next attempted to amplify the DpnI‐resistant replication products by PCR and use next‐generation DNA sequencing (NGS, approach II on Fig. 4B). We performed a first PCR reaction with a 652‐bp product spanning the lesion site and five DpnI sites. This conventional PCR gave no product on DpnI‐digested plasmid from control reactions lacking T antigen, unlike the short qPCR amplicon mentioned above, suggesting that the T antigen independent replication observed in Fig. 4A is inefficient and/or that DpnI has some limited activity on hemimethylated DNA even in the high salt buffer. In our earlier experiments, we showed that a PCR‐based approach can also provide information about postreplicative gaps, because the PCR polymerase is able to bypass the lesion remaining in the only continuous template strand of gapped plasmids. This bypass is not coupled with base insertions; rather, it produces typically 2‐base deletions at the site of the photoproduct [34]. With the NGS approach, the DpnI‐digested replicated products of many experiments can be used as templates in a two‐stage nested PCR extending the DNA with an index sequence for identification (see Materials and methods). When assaying TT(CPD) bypass by NGS (Fig. 4F), we measured a lower contribution of TLS to the bypass spectrum than with the previous *E. coli* system suggesting that *E. coli* really contributed to the former results. TT(CPD) bypass was almost exclusively error‐free, showing the insertion of AA opposite the lesion (Table S3,S4). We also assayed replication on TT(6‐4) lesion and found that TLS deployment is very inefficient with this lesion (Fig. 4F), suggesting that two‐polymerase bypass does not work properly in cellular extract.

Our results agree with earlier findings that CPD lesions are efficiently bypassed by T antigen‐driven replication in an error‐free manner [29, 31], and the introduction of NGS‐based detection delivered detailed information on the very low mutagenicity of TLS over this lesion. The high amounts of replicated DNA make this *in vitro* system especially suited for NGS analysis, which has a clear advantage in throughput compared to Sanger sequencing, and the advantage of finding all arising mutation types compared to hybridisation‐based mutation detection methods.

### Interference approaches: Polymerase η is the main contributor to TLS on TT(CPD) lesions, while the two‐polymerase branch of TLS required for TT(6‐4) lesion bypass is inefficient

**Fig. 5:**
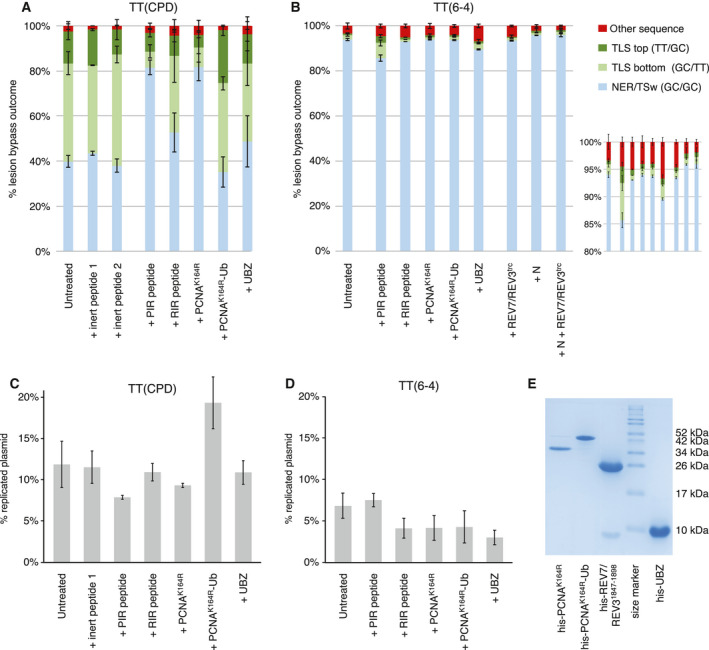
Dissecting the mechanism of lesion bypass using interference assays. (A, B) Proportions of successful deletion‐free TT(CPD) (A) or TT(6‐4) (B) lesion‐bypass outcomes as measured using amplicon sequencing. The replication reactions were supplemented with the indicated peptides or recombinant proteins (100 µm of each peptide, see Materials and methods for all amounts and for details of the control peptides; N, nuclear extract). The mean of three experiments is shown; error bars indicate SD of the column below. The inset in (B) magnifies the top of the columns. (C, D) Replication efficiencies with TT(CPD) and TT(6‐4) lesions. The mean and SEM of three experiments are shown. (E) SDS/PAGE image of the recombinant proteins used in the interference assays.

Next, we employed interference approaches using the addition of peptides and recombinantly expressed protein domains, mutant or fusion proteins to assess the mechanisms of TLS that are active in the extract. We tested the operability of the one‐polymerase system of TLS [8] on TT(CPD) bypass in commercial HeLa extracts using a PIR peptide (also used in a peptide‐binding study [53]) that contains the PIP motif and a part of the NLS sequences plus the last four C‐terminal amino acids of polη, which as we anticipated might interfere with the binding of polη to PCNA. The recombinantly expressed UBZ domain of polη (Fig. 5E) was also employed for its ability to interfere with TLS as expected if TLS is dependent on PCNA ubiquitylation. We planned to block the two‐polymerase TLS system [54] with a RIR peptide corresponding to the REV1 binding domain of polη, as we speculated that this might interfere with the coordination of polη/polκ/polι by the CTD domain of REV1 [55]. Also, by adding a truncated recombinant polζ2 (Fig. 5E) in excess, we expected that it could displace matured polζ4 (REV7‐REV3‐POLD2‐POLD3) from REV1’s C terminus, thereby inhibiting extension. We supposed that the polη‐derived RIR peptide could also compete with the binding of POLD3’s RIR domain reported to be involved in polymerase switch process [56].

The addition of the PIR peptide reduced the deployment of TLS on the TT(CPD) construct (from 57% to 15%, *P* = 0.0029, *t*‐test) and also reduced replication efficiency significantly (*P* = 0.006) while RIR and UBZ peptides had minimal and less significant effects on TLS (*P* = 0.045 and 1.00, respectively) and on replication efficiency (*P* = 0.26 and 0.55, respectively) (Fig. 5A,C). The binding of polη to PCNA is mostly mediated by its PIP‐box [57]. The PIR peptide harbours this element, as well as some part of the NLS sequences and the C‐terminal PLTH amino acids, which were found to also significantly contribute to a specific polη‐PCNA interaction [53]. As the polη‐derived peptide inhibits the observed error‐free and efficient TLS, polη is likely to be the main actor in bypassing TT(CPD) in HeLa extract. Polκ or polι are expected to be less inhibited by this highly specific peptide, as it was shown that their homologous regions show different binding modes and adopt different structures when binding PCNA [53].

TLS polymerases also contain ubiquitin‐binding domain(s) through which they can bind to ubiquitylated PCNA. Although this interaction is weaker (*K*_d_ = 73 ± 13 μm as determined by NMR) [58] than that of submicromolar PIP‐box‐PCNA interactions, the cooperativity of this interaction was found to be important for TLS [59]. In another line of interference studies, we observed a reduction of TLS outcomes on the TT(CPD) lesion upon addition of the PCNAK164R mutant protein on which the target lysine of ubiquitylation is replaced by an arginine (Fig. 5A,E; *P* < 0.001, *t*‐test). As PCNA recruitment from the extract is required for *in vitro* DNA replication [60], this suggests that PCNA ubiquitylation is important for efficient *in vitro* TLS and that this ubiquitylation process is somehow operative in cytosolic extracts. Conversely, the addition of a PCNAK164R‐Ub fusion protein, which efficiently binds Y family polymerases [14], slightly increased TLS deployment (Fig. 5A,E) and significantly increased replication efficiency (Fig. 5C; *P* = 0.021, *t*‐test). As PCNAK164R interference reduced TLS and also slightly reduced replication efficiency on TT(CPD)‐containing templates, our data indicate that TLS in the HeLa extract is primarily controlled through PCNA ubiquitylation. Indeed, PCNA was found to be ubiquitylated in extracts of MRC5 human fibroblasts [61] and COS‐7 cells [62]. Interestingly, TLS was relatively more reduced on the bottom strand upon the addition of the PIR peptide or PCNAK164R interference, raising the possibility that the inhibited TLS pathway is more active on the leading strand. Some negative, but nonsignificant effect of the UBZ peptide on TLS, was also observed, which could be explained by its weak competition with TLS polymerases for binding ubiquitylated PCNA. TLS was not completely inactivated by PIR and PCNA‐K164R, suggesting that alternative TLS polymerases or TLS recruitment mechanisms also contribute to the process. The weaker inhibitory action of RIR peptide (Fig. 5A) suggests that TLS coordinated by REV1 can also contribute to the process. Indeed, cells lacking polη can also bypass TT(CPD) [34, 63]. The TLS polymerase polι was shown to be able to insert nucleotides against TT(CPD) [64], and polκ was suggested to possess extender function [65]. In addition, polθ was recently suggested to be able to insert nucleotides against UV lesions [66], thus can also contribute to the redundancy in TLS on TT(CPD).

As was shown above, TLS bypass of TT(6‐4) lesions is rather inefficient in HeLa extract (Fig. 4F). A similar observation was also made in a former study [30]. The likely explanation is that the bypass of this lesion requires a ‘two‐step’ process with the involvement of the general extender complex for extension from the mismatched primer terminus formed by the insertion step by an ‘inserter’ polymerase [10]. We therefore sought also to test the contribution of the extender complex by designing an interference approach, whereby recombinantly expressed and purified truncated polζ2 (a copurified complex of full‐length REV7 and residues 1847–1898 of REV3) was added in a concentration much higher than the observed Kd (1.3 µm) for the REV1/REV7 interaction [67]. No effect was seen with this interference, nor with the RIR peptide designed also to interfere with the binding of the polζ4 extender to the REV1 CTD domain. This suggests that this process is dysfunctional in HeLa extract or works at low efficiency (Fig. 5B,D). Interestingly, PIR and UBZ peptides had an unexpected and an inverse effect with TT(6‐4) lesion compared to that was observed for TT(CPD) lesion, increasing TLS deployment slightly (inset of Fig. 5B). This might be the consequence of an increased polymerase change capacity, whereby these interferences would facilitate the process by weakening the interaction of the inserter polymerase with PCNA. The general extender complex (polζ4) might be in a limiting amount in the extract (even when supplemented with nuclear extract, Fig. 5B column 8) such that it cannot compete with the inserter. Alternatively, the polη‐directed interferences might facilitate the use of alternative polymerases that are able to execute both insertion and extension steps. Indeed, it was shown that in the lack of Polζ the inefficient TLS on TT(6‐4) lesions was reactivated when polη was also deleted [10], in an analogous situation to that when binding of polη is inhibited and Polζ is dysfunctional in our study.

### NER modulates lesion‐bypass detection

A specific confounder in our bypass studies with HeLa extract was that GC/GC sequence outcomes could also be generated by NER, not only by TSw. NER can remove the lesions during incubation thus removing the substrate for the bypass routes. This might also be a reason why slower processes (as two‐step TLS) are masked. To reveal the contribution of NER, we repeated the above studies using extract from NER‐deficient cells. For this, we employed the TK6 human lymphoblastoid cell line in which a knockout of the key NER gene *XPA* is available together with an isogenic wild‐type control [36].

**Fig. 6:**
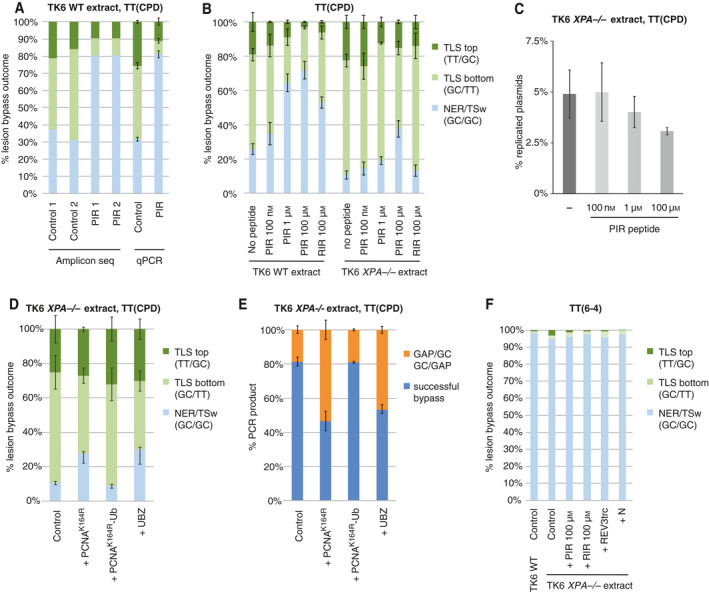
NER contributes significantly to GC/GC outcomes and to successful replication. (A) Lesion‐specific qPCR and NGS give identical results. NGS data from two repeats of control and PIR peptide‐treated CPD replication reactions in TK6 extracts are compared with qPCR measurements (mean and SD of four experiments) of the same experimental conditions. Only correct TLS and GC/GC outcomes are shown. (B) Bypass outcomes upon PIR and RIR peptide interference in NER‐proficient and NER‐deficient TK6 extract. Mean and SD of 3–4 experiments are shown. (C) Decreasing replication efficiency with increasing PIR concentration. (D) TT(CPD) bypass in NER‐deficient TK6 extract under interference by two PCNA variants or the UBZ protein domain. Mean and SD of three experiments are shown. (E) An increase in postreplicative gaps (deletions at the lesion, GAP) in the same experiments as in panel (D) indicates less efficient complete replication in NER‐deficient extracts. (F) TLS on TT(6‐4) containing pUCQF plasmid is as inefficient in TK6 (WT or *XPA* mutant) extracts as in HeLa extract. No significant effects were seen in replication outcomes with the indicated interference approaches. For all panels, peptides and proteins were added at concentrations specified in the Materials and methods unless otherwise indicated.

In order to speed up and simplify the evaluation of the experiments, we established a sequence‐specific qPCR assay (approach III on Fig. 4B) which enabled us to detect the common sequence outcomes detected by NGS: the main bypass and repair products (TLS, TSw/NER) and the shorter PCR products (GAPs) indicative of postreplicative gaps (Fig. 4G). In this assay, we used combinations of sequence‐specific primers to measure the abundance of each main outcome (GC/GC, TT/GC, GC/TT, GAP/GC, GC/GAP). The re‐analysis of existing samples with qPCR provided extremely similar results to those obtained with NGS (Fig. 6A).

We compared the replication products of TT(CPD)‐containing plasmids incubated in extracts of either the wild‐type (WT) or *XPA* knockout TK6 cells. Using the qPCR method, we measured 10% GC/GC outcomes in the XPA‐deficient extract as opposed to 26% in the wild‐type control (Fig. 6B), suggesting that the majority of GC/GC sequences were the result of NER repairing the photoproduct using the GC sequence on the opposite strand as template prior to replication, rather than template switch during replication. In agreement with this, in the NER‐deficient extract we observed a gradual reduction of the proportion of TLS among replicated sequences when we added increasing concentrations of the PIR peptide (Fig. 6B). In the wild‐type extract, the inhibition was near saturation at 1 µm concentration, but only a slight inhibition was observed with 100 nm peptide. A different pattern was seen with NER‐deficient extract (right panel). In this case, lesions are not removed during incubation, potentially providing more time for alternative, but slower TLS pathways to bypass lesion even if the main route by polη is blocked. Although TLS was only barely inhibited at 1 µm peptide concentration, it was substantially inhibited at 100 µm (Fig. 6B) with a significant decrease in replication efficiency (Fig. 6C, *P* = 0.025, *t*‐test), suggesting the partial inhibition of the alternative TLS pathways too. The RIR peptide caused only moderate TLS inhibition in both TK6 WT and TK6 *XPA* *^−/−^* extracts, similar to what was observed in the HeLa extract, suggesting at most a minor contribution of REV1‐dependent processes to TLS on the TT(CPD) lesion.

PCNAK164R and PCNAK164R‐Ub interference experiments were also repeated in a NER‐deficient background. We observed a similar trend to that found with HeLa extract, but with much smaller changes to the proportion of TLS outcomes (Fig. 6D). In addition to reducing the proportion of TLS among successful bypass products, PCNAK164R and UBZ treatment also increased the proportion of GAP qPCR products (Fig. 6E). As we demonstrated before, these products can arise during synthesis across the lesion by the PCR polymerase, indicating that upon downregulation of TLS in NER‐deficient extracts through interference with PCNA ubiquitylation, a larger proportion of the recovered replicated plasmids contain the lesion in an unreplicated single‐stranded region [29]. The lower number of gapped products and the higher proportion of GC/GC outcomes in the wild‐type extract therefore must be a consequence of active NER removing the TT(CPD) lesion prior to replication.

Fused ubiquitin (PCNAK164R‐Ub) had no significant effect on TLS deployment and GAP formation (Fig. 6D,E), suggesting that PCNA ubiquitylation is already efficient in the assay.

On the TT(6‐4) photoproduct, we observed almost as inefficient TLS bypass in TK6 wild‐type and XPA‐deficient cell extracts as in the HeLa extract. Some of the interferences were also tested here showing no significant effect on the bypass routes (Fig. 6F). In the absence of both TLS and NER, nearly all recovered sequences showed GC/GC, which could be the product of TSw activity or a PCR artefact (see below).

### NER can readily be investigated in cellular extracts, but the detection of template switching is compromised by a PCR artefact

**Fig. 7:**
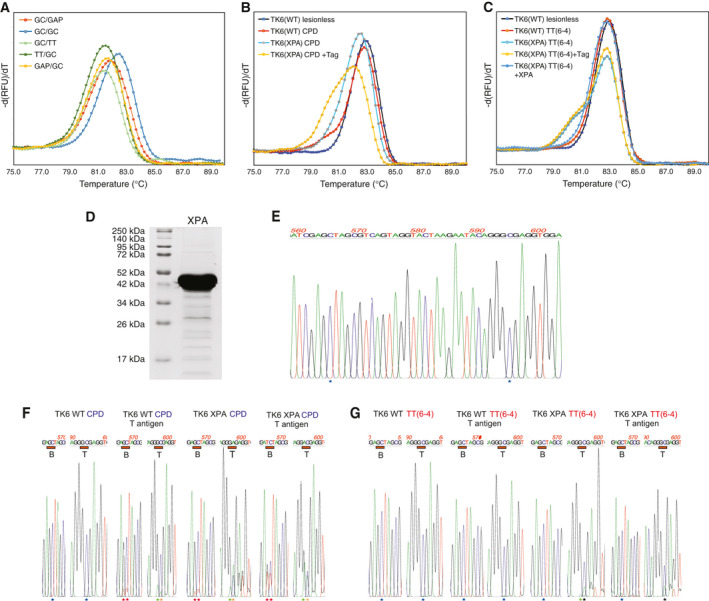
NER can remove both TT(CPD) and TT(6‐4) photolesions during incubation in cellular extract. (A) Control melting curves of PCR products generated from template DNAs with different individual lesion‐bypass sequences (GC/GAP, GC/GC, GC/TT, TT/GC, GAP/GC) at the lesion sites. (B, C) Melt curves of 140 bp PCR products generated by nested PCR on TT(CPD) templates (B) and TT(6‐4) templates (C) recovered from replication reactions in the absence or presence of T antigen. A complex curve was observed in the presence of T antigen (yellow) with TT(CPD) material giving rise to many TLS events, whereas a simple curve (red) similar to that obtained with the lesionless material (black) was generated with the NER‐proficient extract on both lesions. Curves obtained with templates from the NER‐deficient *XPA* *^−/−^* extract (blue) are different from those obtained with lesionless constructs, suggesting a failure of lesion removal. Recombinantly expressed and purified XPA complemented the *XPA* *^−/−^* extract (dark blue line). (D) SDS/PAGE image of the purified recombinant XPA protein preparation used in (C). The experiments were performed several times; representative melting curves are shown in panels (A, B, D). (E) Sequencing chromatogram of the lesionless control plasmid, with the sites of the lesions marked with asterisks. (F, G) Short sections of sequencing chromatograms of bulk lesion‐containing plasmid DNA prepared after incubation in TK6 NER‐proficient (WT) or NER‐deficient (*XPA* *^−/−^*) extracts in the absence or presence of T antigen as shown. The lesion sites are marked with red bars below the sequence (B, bottom strand; T, top strand). Double peaks indicating shorter and longer PCR products are seen on the sequencing chromatograms in NER‐deficient conditions at the top lesion site in case of both CPD and 6‐4 photoproducts. Almost perfect sequences with GC (blue asterisk) at the lesion sites are seen in the NER‐proficient extracts. Signs of TLS (marked with red and green asterisks) are observed with TT(CPD) lesion in XPA extracts.

As NER‐proficient and NER‐deficient extracts showed different bypass dynamics, we investigated the capacity of NER for removing UV photolesions during the 3‐h incubation time in the absence or presence of T antigen. We omitted DpnI digestion here to keep nonreplicated material also intact. The extent of NER as well as replication‐coupled TLS could be directly detected by Sanger sequencing of an amplified 652 bp part of the plasmid encompassing the lesion sites and comparing to the sequencing profile of a lesionless construct with GC at the lesion site (approach IV, without DpnI digestion, Fig. 4B). In the wild‐type extract, the signal showed the GC sequence at the lesion site similarly to that found with the lesionless construct (Fig. 7E,F, blue asterisks), suggesting efficient repair by NER using the opposite strand as template. In contrast, we observed the appearance of a shorter PCR product (double peaks downstream of the lesion site) in case of both TT(CPD) and TT(6‐4) lesions following incubation in the NER‐deficient TK6 *XPA* extract (Fig. 7G), indicating that the lesion was not removed by NER. The shorter PCR products (mostly formed at the top lesion site) are presumably generated by the Pfu PCR polymerase when it skips unremoved lesions in the template.

Interestingly, some TLS also appeared in the absence of T antigen in the case of TT(CPD) at both top at bottom lesion sites, especially in the NER‐deficient extract, which may be the result of some basal replication activity of the extract itself. However, considerably more TLS products formed in the presence of T antigen. The above findings also can be observed on the melting curves recorded at the end of a PCR run amplifying the 140‐bp lesion‐containing part of the template used also for Sanger sequencing (Fig. 7). In the absence of T antigen, PCR products of lower melting temperature appeared in NER‐deficient extracts than in WT extracts (Fig. 7B light blue vs red curves), indicating more TLS activity in the NER‐deficient extract (compare to standard curves in Fig. 7A). In the case of NER‐proficient extract, little difference was seen between the lesionless and CPD‐containing samples (Fig. 7B, dark blue and red curves) as TLS bypass could not compete with the efficient lesion removal.

A simple melting curve resembling that of the lesion‐free material was obtained with the TT(6‐4) material in the case of NER‐proficient extracts (Fig. 7C, red curve), indicating complete removal of the lesion, while nonremoval in XPA‐deficient extract was reflected in double curves (Fig. 7C, light blue and yellow) both in the presence and in the absence of T antigen with a lower temperature peak representing the shorter PCR products (gapped plasmids) forming when Pfu polymerase ‘jumps’ over the lesion. Recombinantly expressed and purified XPA (Fig. 7D) was able to complement the missing NER activity in XPA‐deficient cellular extract, manifest in the appearance of simple melting curve equivalent to that obtained with the lesionless plasmid (Fig. 7C). No evidence of effective TLS was seen here even in the presence of T antigen, as its inclusion had no effect on the melting curve. The similarly high GAP content observed with or without T antigen also suggested inefficient TSw.

**Fig. 8:**
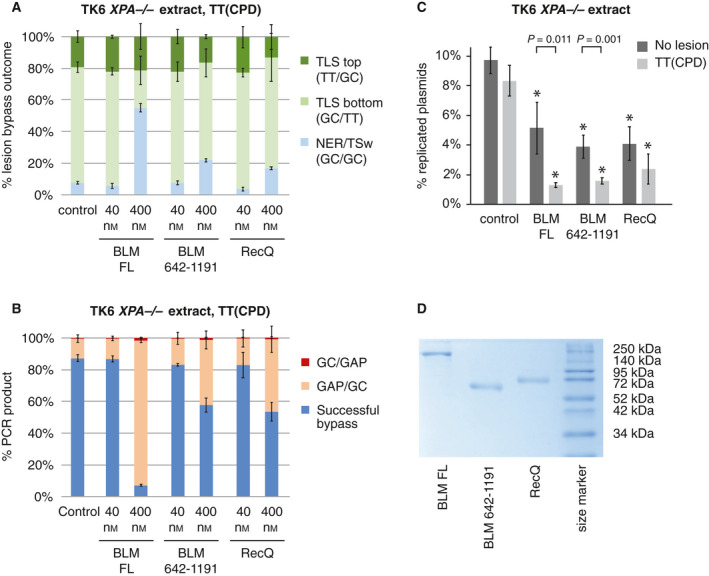
RecQ helicases inhibit *in vitro* replication. (A, B) BLM helicase variants (FL, full length) or the bacterial RecQ helicase increase the proportion of GC/GC bypass outcomes (A, light blue) as well as GAP formation (B, orange and red) at high concentration, as detected by sequence‐specific qPCR. The mean and SD of three experiments are shown. (C) Replication efficiency in replication reactions upon the addition of recombinant BLM variants or RecQ at 400 nm concentration. The mean and SD of three experiments are shown. Significant differences to the control reaction with the same template are indicated with asterisks (*P* < 0.01, *t*‐test); template‐specific statistical differences are shown on top. Increased GAP content is associated with lower replication efficiency and increased GC/GC outcomes. (D) SDS/PAGE image of the recombinant helicases used in the assays. * are mentioned as asterisks.

To test whether GC/GC outcomes in XPA‐deficient extracts represent real TSw events, we attempted an interference approach using recombinant human BLM helicase or bacterial RecQ helicase (Fig. 8D), both potentially capable of disrupting D‐loops [43, 45] and thus acting against TSw that requires the formation of this intermediate. When we added either helicase to replication reactions with TT(CPD), no significant effect was seen at low (40 nm) concentration. However, we observed a drastic increase in GAPs at higher BLM helicase concentration (400 nm, amounting to about 1.9% of the total protein in the assay), and a similar, but moderate increase with the less active BLM core domain or RecQ (Fig. 8B). The proportion of GC/GC outcomes also increased, mirroring the lower rates of successful replication (Fig. 8A). Indeed, the replication efficiency decreased considerably upon the addition of the BLM versions or RecQ (Fig. 8C), suggesting that the helicases primarily interfere with replication, rather than with TSw. The increased relative GC/GC signal could be caused by an artefactual generation of PCR hybrids, whereby the polymerase does not always jump through the lesion generating a small deletion (GAP), but stops; then, the partially amplified fragment aligns with the complementary fragment generated at the other lesion site to prime further amplification. Artefact formation is likely negligible in cellular extract of a NER‐proficient cells as NER generates easily amplifiable material but can be an issue with TT(6‐4) lesion bypass in NER‐deficient cellular extracts where both TLS and TSw are inefficient. The suspected formation of PCR hybrids hampers the identification of true TSw events that would also generate GC/GC outcome. BLM helicase caused a significantly greater reduction of replication efficiency on the lesion‐containing template, which is suggestive of a specific inhibition of lesion bypass that may be TSw (Fig. 8C). Nevertheless, the predominance of TLS on TT(CPD) in XPA‐deficient extracts, and the appearance of postreplicative gaps under all conditions that inhibit TLS, suggests that there is no more than a minor TSw activity in the *in vitro* system.

## Conclusions

We have demonstrated that T antigen‐driven plasmid replication in cytosolic extracts of wild‐type or genetically modified cells is suitable for detecting and investigating TLS and that the same *in vitro* assays provide evidence of rapid and efficient NER. Furthermore, we established and described a range of state‐of‐the‐art methods for analysing the outcome of lesion bypass and DNA repair. Although our *in vitro* assay has a limitation in reproducing and detecting true TSw bypass events, it turned out to be suitable for testing TLS both in the presence and in the absence of active NER. TLS on TT(CPD) lesions is mainly achieved by polη, potentially in redundancy with other Y family polymerases. The observed PCR‐related deletions, readily detected by NGS or sequence‐specific primer pairs, provide a quantitative measure of replicated plasmids which failed lesion bypass.

Our main aim was to test the applicability of T antigen‐driven *in vitro* replication for easily performable interference studies with exogenous agents and to develop or adapt modern assay methods to classic experiments for the simultaneous detection of multiple DNA damage bypass and repair processes. The use of a high‐throughput NGS readout of lesion bypass indicated near‐perfect error‐free TLS over TT(CPD) photoproducts. Our data are in line with the concept that PCNA ubiquitylation enhances TLS. The dependence of CPD bypass on PCNA ubiquitylation but not polζ suggests that the mutagenic TLS over CPD lesions observed in living cells [34] is due to two‐polymerase bypass activity that is largely lacking in the *in vitro* system. The inverse effects of PIR peptide and UBZ peptide on TT(6‐4) bypass might suggest a facilitated polymerase switch mechanism, but more studies are required on this topic. NER was very efficient in TK6 and HeLa cellular extracts; thus, this methodology can be used for testing the removal of lesion‐forming toxic substances that cannot readily enter cells. The comparable contributions of nonmutagenic NER and potentially mutagenic TLS to the sequence outcome of UV lesions emphasise the relevance of cellular proliferation rate to the mutagenicity of DNA‐damaging agents. Some modifications of the established methodology would still be required to eliminate the formation of PCR artefacts and improve the specific detection of potential recombination‐derived bypass products.

## Conflict of interest

The authors declare no conflict of interest.

## Author contributions

ZS and DS conceived and designed the project, ZS, ÁP and GMH acquired the data, ZS, ÁP, GMH, MK and DS analysed and interpreted the data, DS and MK supervised the project, ZS and DS wrote the paper. All authors have read and agreed to the published version of the manuscript.

## Supporting information


**Table S1.** Specificities of sequence‐specific primer pairs.
**Table S2.** Sequence outcome of T antigen driven replication reactions.
**Table S3.** Read counts of all detected sequences at the pUCQF lesion sites.
**Table S4.** Percentages of all detected sequences at the pUCQF lesion sites.

## Data Availability

All primary data are available from the corresponding author upon request.
